# Asthma and hypoxia

**DOI:** 10.1186/1824-7288-41-S2-A55

**Published:** 2015-09-30

**Authors:** Piero Pavone, Raffaele Falsaperla

**Affiliations:** 1 Unità Operativa di Pediatria e Pronto Soccorso Pediatrico, Azienda Ospedaliera Universitaria “Policlinico-Vittorio Emanuele”, Catania, 9100, Italy

## 

More or less severe asthma attacks can damage the CNS and peripheral system in adults and children, both directly causing hypoxia and indirectly with the production of autoimmune antibodies.

The pathogenic mechanism is still unknown; cytokines, TNFs, interleukins, free radicals and nitric oxide are often involved as causative factors for the disorders [1,2].

In this review we focus our attention on the relationship between atopy and CNS (central) or PNS (peripheral system) involvement.

The main disorders associated with atopic disease are:

Atopic myelitis

Hopkins syndrome

Hirayama disease

Spinal progressive muscular atrophy (SPMA)

The occurrence of myelitis in patients with atopic diathesis (atopic myelitis) affecting young adults has been widely reported especially in Japan.[2,3]

In general, the disorders affect the posterior column of the cervical spinal cord, as shown clinically and by an MRI, and are characterized by hyperIgEaemia and the presence of light antigen-specific IgE. Another disease that has been reported in association with atopic disorders is Hirayama disease, a juvenile muscular atrophy of the distal upper extremity, which is also associated with airway allergies such as allergic rhinitis and atopic asthma.

Our group reported on a 13-year-old girl who had recurrent acute episodes of myelitis after asthma attacks; the patient showed to be affected by Hopkins syndrome [4] (HS); a flaccid paralysis affecting one or more limbs after an asthma attack. It is a poliomyelitis-like illness, with frequent recurrences in which a possible link between the disease and atopic myelitis was reported, but until now the precise etiopathogenetic mechanism still remains unknown.

Prospective study on the history of allergic disorders in known neurological diseases, an association between spinal progressive muscular atrophy and asthma as well as between myelitis and atopic dermatitis has been demonstrated.

Central nervous system damage associated with atopic diathesis may be classified into two categories: eosinophilic myelitis mainly affecting the cervical spinal cord and those lower motor neuron, such as Hopkins syndrome, Hirayama disease and SPMA [2,4].

Recently we reported children with epileptic crisis related to cow's milk protein allergy; this disease can manifest itself with skin reactions, failure to thrive, and anaphylaxis as well as gastrointestinal and respiratory disorders [5].

In conclusion, the aim of this report is to analyse current data on the link between asthma and CNS/or PNS involvement and also, cow's milk protein allergy and epileptic events, highlighting the presence and scientific evidence for any potential pathogenetic mechanism.
